# Infectious diseases and childhood disruptive behavior disorders: a reappraisal

**DOI:** 10.3389/fcimb.2026.1754005

**Published:** 2026-03-12

**Authors:** Ravi Philip Rajkumar

**Affiliations:** Department of Psychiatry, Jawaharlal Institute of Postgraduate Medical Education and Research, Pondicherry, India

**Keywords:** autoimmunity, basal ganglia, childhood infectious diseases, conduct disorder, disruptive behavior disorders, gastroenteritis, gut-brain axis, oppositional defiant disorder

## 1 Introduction

Disruptive behavior disorders (DBDs) of childhood include conduct disorder (CD) and oppositional defiant disorder (ODD). These conditions are characterized by recurrent defiant, disruptive, or aggressive behaviors that go against accepted social norms (Robles et al., 2022). ODD is characterized by defiance towards authority figures, often accompanied by chronic irritability or anger outbursts. CD is a more severe disorder characterized by prominent rule-breaking, disregard for the rights or feelings of others, and prominent aggression or violence (Fairchild et al., 2019). ODD may represent a milder form of CD, or a distinct condition that overlaps clinically and etiologically with it (Blair et al., 2014).

DBDs are considered to arise from complex gene-environment interactions. Genes implicated in DBDs include those related to the monoamine neurotransmitters serotonin and dopamine, the hormones testosterone and cortisol, and the neuropeptide oxytocin (Holz et al., 2018; Moore et al., 2019). These interact with environmental factors such as prenatal exposure to alcohol, tobacco or viral infections, peri-or neonatal complications, early childhood adversity, parental exposure to stress, and parenting style (Latimer et al., 2012). This results in alterations in the structure and functioning of brain circuits connecting the frontal, anterior cingulate and temporal cortices to the amygdala, caudate nucleus, and thalamus. These circuits are involved in psychological processes such as decision-making, responsiveness to threats, empathy, emotional regulation, and social behavior. Dysfunction of these circuits leads to traits of irritability, callousness and aggression. These manifest as symptoms of ODD or CD at home, in school, and in various social settings (Jansen, 2022; Gao et al., 2024).

DBDs are associated with significant individual and social costs across the life-span. In childhood, these include conflicts with parents, teachers, and the juvenile justice system, low educational attainment, and substance misuse. Adult consequences of untreated DBDs include substance use disorders, unemployment, criminality, intimate partner violence, child abuse, and suicide (Blair et al., 2014; Fairchild et al., 2019; Goulter et al., 2023). Individual and family therapies have the best evidence of efficacy in the management of DBDs. These treatment approaches are not always available, particularly in low- or middle-income settings, and response to treatment is often incomplete (Perlstein et al., 2023). Pharmacological agents, particularly antipsychotics, have modest effects on symptoms of aggression and irritability. The long-term efficacy and safety of these drugs is unknown (Loy et al., 2017). For these reasons, the identification of novel approaches for the prevention and treatment of DBDs is a priority area in child and adolescent mental health (Waddell et al., 2007; Hofvander et al., 2017).

Environmental risk factors account for 40-60% of the variation in the risk of DBDs (Azeredo et al., 2018). Most research to date has focused on psychosocial risk factors, such as harsh or inconsistent parenting practices, parental mental illness or substance use, school-related problems, and socioeconomic disadvantage (Murray et al., 2013; Lin et al., 2022a; Liu, 2025). However, relatively less is known about the role of other factors that could affect brain development *in utero* or in early childhood, such as perinatal insults, maternal substance abuse during pregnancy, allergic disorders, or childhood infections (Latimer et al., 2012; Elbagir et al., 2023). Research over the past decade, especially in low- and middle-income countries, has reported high rates of DBD symptoms in older children and adolescents with past or current infections (Kamau et al., 2012; Chandna et al., 2022). As this is a potentially modifiable risk factor, it is important from both clinical and public health perspectives to know whether there is a genuine causal link between childhood infections and DBDs. The purpose of this paper is to briefly examine the plausibility of such a link through a careful examination of the available data.

## 2 Methods

A preliminary literature search revealed that the available research on this topic was heterogeneous and did not lend itself to a formal systematic review. Therefore, a narrative synthesis and conceptual analysis of available results was undertaken, in keeping with standard recommendations (Gregory and Denniss, 2018; Sukhera, 2022). The PubMed, ScienceDirect and Scopus databases were searched using the keywords “infection”, “infectious”, “infective”, or “infectious disease”, both alone or in conjunction with “respiratory”, “gastrointestinal”, or “childhood”, along with the keywords “oppositional defiant disorder”, “conduct disorder”, or “disruptive behavior disorder”, up to and including the month of December 2025. Papers were selected for discussion only if they reported the results of original research, case reports, or case series examining the association between infectious diseases and childhood DBDs, either in general or in relation to specific pathogens. Out of 310 initial citations screened, a total of 58 relevant articles were selected.

The key findings and hypotheses outlined in these papers were summarized under three headings:

- General associations between infectious diseases and DBDs in childhood
- Associations between specific infections or pathogens and DBDs in childhood
- Possible mechanisms linking infection to the onset of pediatric DBDs or DBD symptoms

Following this, the available evidence was critically appraised and synthesized to address any possible confounding effects, or limitations of the existing literature.

## 3 Results

### 3.1 Disruptive behavior disorders and infection: pre- and postnatal associations

A risk factor that merits further investigation in this context is exposure to infectious pathogens early in life. Prenatal viral infection has been considered a possible risk factor for childhood DBDs (Latimer et al., 2012). Maternal systemic inflammation, as indexed by elevated serum C-reactive protein (CRP), is not directly associated with DBDs (Chudal et al., 2020). On the other hand, specific histological evidence of chorionic vasculitis and umbilical cord inflammation, known as the fetal inflammatory syndrome (FIRS), is associated with an approximately 1.5-fold increase in the risk of CD. The FIRS can be induced by several factors, including viral infection (Gibson et al., 2023). Maternal respiratory infections have been associated with an elevated risk of attention-deficit/hyperactivity disorder, a neurodevelopmental condition that is highly comorbid with DBDs (Pineda et al., 2007).

These findings naturally lead to the question of whether post-natal infections, particularly in early childhood, are also associated with DBDs. A study of pre-term infants with confirmed or suspected neonatal infections, followed up till the age of 9 years, found evidence of an association with ADHD, but not with DBDs across the three groups. However, confirmed neonatal infection was associated with a nearly two-fold increase in CD frequency (Rand et al., 2016). In contrast, exposure to infection later in childhood may be associated with CD or ODD. In a study which examined over 1,000,000 Danish children until a mean age of 9.8 years, infections requiring either hospitalization or treatment with antibiotics were associated with a 1.5- to 3-fold increase in subsequent CD or ODD (Köhler-Forsberg et al., 2019). A similar study of 1,285 youth from the United States found that infections requiring antibiotic therapy during the first year of life, as reported by parents or caregivers, was associated with a 3.7-fold increase in ODD at ages 9 to 17; the number of children diagnosed with CD was too small for a meaningful comparison (Goodwin, 2011). A longitudinal study of children with fever of unknown origin did not report significant associations with DBDs, but this study included several children with non-infectious causes of fever, and did not include a control group (Weakley et al., 2020).

### 3.2 Are specific infectious pathogens associated with disruptive behavior disorders?

The next question that arises is whether the association between infections and DBDs is non-specific, or whether certain pathogens are associated a particular increase in risk. Infections that directly affect the brain have been associated with the emergence of CD- or ODD-like symptoms in previously healthy children: in these cases, the symptoms are probably the consequence of direct brain damage caused by the pathogen, and are often accompanied by prominent cognitive impairment. Such presentations were first documented during the encephalitis lethargica outbreak of the 1920s, in which some affected children were reported to exhibit irritability and aggressive behavior similar to that seen in CD, and which represented a radical change from their premorbid selves (Cheyette and Cummings, 1995; Ruiz, 2015). Similar presentations have been reported more recently in children with acute viral encephalitis (Deka et al., 2006; Lin et al., 2022b; Bergman et al., 2024), cerebral malaria (Sveinbjornsdottir et al., 2025), and chronic encephalitis caused by the Epstein-Barr virus (EBV) (Caruso et al., 2000). Though these cases cannot be considered “true” DBDs, they may provide valuable clues about the functional neuroanatomy and neurophysiology of DBDs, as discussed in a subsequent section. Leaving these aside, the following associations between specific infections and DBDs in childhood have been documented in the literature:

#### 3.2.1 Human immunodeficiency virus and acquired immunodeficiency syndrome

Perinatal infection with HIV has been associated with subsequent CD or ODD in youth in several studies from the United States. In a sample of youth with perinatal HIV infection, currently aged 9–16 years, 13% were diagnosed with CD and 11% with ODD. The majority of this sample were receiving antiretroviral treatment (ART) and more than 90% had CD4+ counts higher than 200 cells/mm3, indicating relatively well-preserved immune function (Mellins et al., 2006). Markers of HIV disease severity, such as CD4+ counts less than 660 cells/mm3 or low CD4+ counts at baseline, were associated with more severe symptoms of CD (Nozyce et al., 2006; Nachman et al., 2012). However, some researchers failed to find an association between perinatal HIV and DBDs (Gadow et al., 2010), while others found that variations in CD in children with AIDS were more significantly correlated with measures of parent-child attachment and consistency of parental discipline (Osigwe et al., 2020; Drabick et al., 2025). These associations are not mutually exclusive: it is highly likely that both disease activity and social factors play a role in the pathogenesis of DBDs in HIV-positive youth.

Results from other countries yield a slightly different picture. Among children and adolescents from India and Uganda, 5-9% were diagnosed to have either CD or ODD, and these conditions were more common in younger children. In Kenya, 12% were diagnosed with DBD and 4.5% with CD. Overall, 45-55% of psychiatric diagnoses made in these children were DBDs (Kamau et al., 2012; Pilania et al., 2020; Taylor Salisbury et al., 2020). These studies did not include a healthy control group or examine the association between HIV disease activity and symptoms of ODD or CD. A study of adolescents living with HIV from South Africa found that self-reported health was negatively associated with symptoms of CD. The same study found that these symptoms were significantly associated with psychosocial variables, including HIV-related stigma, experiences of abuse or bullying, the availability of social support, and the parent-adolescent relationship (Boyes et al., 2019).

#### 3.2.2 Intestinal infections

A longitudinal study of children from England, followed up until adolescents, found that episodes of gastroenteritis in the first three years of life were associated with symptoms of CD in both the fourth and seventh years of life. There was an apparent “dose-response” relationship, in that the number of such episodes was correlated with the severity of CD symptoms. At 15 years of age, gastroenteritis was associated with an increased likelihood of being formally diagnosed with either CD or ODD. Though this association was small in magnitude, it was significant even after adjusting for potential confounders. Peripheral levels of the inflammatory markers C-reactive protein (CRP) and interleukin-6 (IL-6) at ages 9 or 15 did not appear to mediate this association (Parent et al., 2019).

Though this result awaits replication, a recent Mendelian randomization study found evidence that the composition of the gut microbiome is associated with CD. More specifically, the bacterial genus *Coprococcus* and class *Coriobacteriia* were associated with a two-fold increase in CD risk, while the genera *Adlercreutzia* and *Ruminococcaceae*, the class *Negativicutes* and the order *Selenomonadales* reduced the risk of CD by approximately one-half (Fatoba and Simpson, 2025). This result is relevant given that both intestinal infections, and the antibiotics, used to treat them can alter the composition of the gut microbiome in childhood (He et al., 2025).

#### 3.2.3 Pediatric acute-onset neuropsychiatric syndrome

Pediatric autoimmune neuropsychiatric disorders associated with streptococcal infections (PANDAS) refer to new-onset behavioral and movement disorders occurring after infections with group A β-hemolytic streptococci. More recently, the concept of PANDAS has been extended to neuropsychiatric symptoms occurring after other infections. This has been referred to as pediatric acute-onset neuropsychiatric syndrome (PANS) (La Bella et al., 2023; Lojek and Rzeszutek, 2025). ODD has been specifically associated with PANDAS and not with other movement disorders of childhood and adolescence (Ben-Pazi et al., 2011), while 27.5% of children with movement disorders following streptococcal pharyngitis have been reported to develop DBDs (Dale et al., 2004). A more recent study using a data mining approach found that ODD symptoms were one of the “core” features of PANS, occurring in 89% of affected children. However, this study did not find any associations between ODD and immune parameters, such as elevated antistreptolysin O (ASO) titer or altered natural killer (NK) cell count. 82% of the children in this study had a history of recurrent respiratory or skin infections (Gagliano et al., 2020). In contrast, a study of older children and adolescents receiving psychiatric out-patient care found that 70% of patients with DBDs had an elevated ASO titer, even in the absence of a diagnosis of PANDAS or PANS (Pozzi et al., 2015).

A recent study conducted across five low- and middle-income countries examined young children (mean age 7.2 years) who had suffered from invasive Group B streptococcal infections – either meningitis or sepsis – in infancy. There were trend-level associations between exposure to this infection and both DBD symptoms and aggressive behavior (*p* = .06 and *p* = .051 respectively) (Chandna et al., 2022). These children were also at an increased risk of depressive symptoms. A plausible interpretation of these results is that streptococcal infection itself may be associated with DBD risk if it occurs early in life and affects infant brain development, whereas streptococcal infection in later life may trigger DBD predominantly through immune mechanisms.

#### 3.2.4 Other infections

Evidence for a link between other types of infection and childhood DBDs is largely anecdotal. In a study of 375 children and adolescents from Iran who were hospitalized with COVID-19, found that nine children (2.4%) developed new-onset ODD, out of a total of 58 with new-onset psychiatric diagnoses; in other words, ODD accounted for 15.5% of new psychiatric diagnoses following COVID-19. The mean age of children receiving this diagnosis was 8.5 years, and it was not significantly associated with either demographic variables or the presence of neurological disorders secondary to COVID-19 (Zahed et al., 2023). In a series of seven individuals who had concurrent infection with two zoonotic infections – the protozoan *Babesia odocoilei* and the bacterium *Bartonella henselae* – one patient was diagnosed with ODD at the age of 9, and subsequently developed depression in adult life (Maggi et al., 2024). On the other hand, a serological study found no association between antibodies to herpes simplex virus (HSV) and conduct disorder in childhood (Sylvester Jorgensen et al., 1982).

## 4 Exploring the mechanisms linking childhood infections and disruptive behavior disorders

The above data, summarized in **Table 1** above, suggests that certain infections may play a role in triggering DBDs in childhood. In the case of HIV/AIDS, it could be argued that these symptoms result from the stigma and psychosocial adversities associated with the disease. However, this does not explain the occurrence of CD or ODD symptomatology in relation to other common infections of childhood. Based on the available evidence, there are at least three plausible mechanisms that may link specific infections to DBDs in the pediatric population.

**Table 1:** Research examining the association between infectious diseases and disruptive behavior disorders in children and adolescents.

Infectious diseases in general		
Study and country of origin	Study design and sample description	Results
Goodwin, 2011 United States	Retrospective analysis of data on 1,279 children and adolescents (age 9–17 years) with or without documented “severe infection in the first year of life”	Severe infection in early life present in 14 of 1279 children. 3 (21%) were diagnosed with ODD. OR for ODD in those exposed to infection was 3.7 (*p* <.05).
Rand et al., 2016 New Zealand	Cohort study of 110 “very preterm” infants (gestational age ≤ 32 weeks) followed up till 9 years or age with either confirmed or suspected neonatal infection.	Confirmed infection in 25%, suspected infection in 23% children. Frequency of CD 11.5% in confirmed infection, 4.2% in suspected infection, 5.8% in those with no infection.
Kohler-Forsberg et al., 2019 Denmark	Register-based cohort study of 1,098,930 children and adolescents (mean age 9.8 years) with a history of either hospitalization for infection or prescription of antibiotics.	34.8% of children or adolescents with CD/ODD had a prior history of hospitalization for infection, and 99.2% had received antibiotics. OR for CD/ODD was 3.2 in those hospitalized for infection, and 2.1 in those requiring antibiotics.
Human immunodeficiency virus (HIV) infection		
Study and country of origin	Study design and sample description	Results
Mellins et al., 2006 United States	Cross-sectional study of 47 children and adolescents (age 9–16 years) with perinatally acquired HIV.	CD present in 13% of patients; ODD present in 11% of patients. No association between recent HIV RNA or CD4+ count and diagnoses of CD or ODD.
Nozyce et al., 2006 United States	Cross-sectional study of 274 children and adolescents (age 2-17) with prior treatment for HIV/AIDS	Significant CD symptoms present in 16% of patients. CD4+ count < 660 cells/mm^3^ significantly associated with CD symptoms.
Gadow et al., 2010 United States	Case-control study of 319 children and adolescents with perinatally acquired HIV (mean age 13.1 years) and 256 controls with HIV exposure or an affected family member, but no infection (mean age 11.3 years).	ODD present in 5% of cases and 7% of controls; CD present in 1% of cases and 3% of controls. No significant difference in the rates of CD or ODD across groups.
Zeegers et al., 2010 South Africa	Cross-sectional study of 100 children (median age 8 years) with HIV/AIDS.	ODD present in 12% of patients. Trend towards a positive association between CD4+ counts and ODD (*p* = .07)
Kamau et al., 2012 Kenya	Cross-sectional study of 162 children and adolescents with HIV/AIDS (age 6–18 years)	ODD present in 12% of patients; CD present in 4.5% of patients.
Nachman et al., 2012 United States	Cross-sectional study of 317 children and adolescents (age 6–17 years) with perinatally acquired HIV.	DBDs present in 14% of patients. Trend (*p* = .09) towards an association between lower CD4+ count and ODD diagnosis.
Osigwe et al., 2020 United States	Cross-sectional study of 314 children and adolescents (age 6–17 years) with perinatally acquired HIV.	Caregiver-reported CD symptoms seen in 9-42% of patients and ODD symptoms in 20-63% of patients.
Pilania et al., 2020 India	Cross-sectional study of 101 adolescents (age 10-17) with HIV/AIDS	ODD present in 6% and CD in 3% of patients.
Taylor Salisbury et al., 2020 Uganda	Cross-sectional study of 1,339 children and adolescents (mean age 10.2 years) with HIV/AIDS.	ODD or CD present in 7% of patients.
Streptococcal infection		
Study and country of origin	Study design and sample description	Results
Dale et al., 2004 United Kingdom	Cross-sectional study of 40 children and adolescents (mean age 7.3 years) with post-streptococcal movement disorders.	ODD present in 15% and CD in 12.5% of affected children. Prevalence of ODD and CD significantly higher than the general population estimates for the United Kingdom (*p* <.001 for both disorders).
Pozzi et al., 2015 Italy	Cross-sectional study of 31 children and adolescent with DBDs (mean age 11.2 years).	70% of patients with DBDs had an elevated ASO titer.
Gagliano et al., 2020 Italy	Artificial neural network-based data mining analysis of clinical and laboratory data for 39 children with PANS (mean age 8.6 years).	89% of patients with PANS had ODD symptoms. No associations between ODD symptoms and ASO titer or NK cell counts.
Chandna et al., 2022 Argentina, India, Kenya, Mozambique, South Africa	Multi-country cohort study of 573 children (mean age 7.2 years), 156 of whom had invasive group B streptococcal infection (sepsis or meningitis) in infancy.	Trend towards associations between early invasive streptococcal infections and both overall DBD symptoms (*p* = .06) and conduct symptoms (*p* = .051).
COVID-19 infection		
Study and country of origin	Study design and sample description	Results
Berloffa et al., 2023 Italy	Case series of 10 children (mean age 9.7 years) with PANS following COVID-19.	70% of children with PANS had ODD symptoms. Three doses of monthly oral pulse prednisolone (1–2 mg/kg for 5 days) resulted in significant improvement symptoms of PANS, including ODD symptomatology.
Zahed et al., 2023 Iran	375 children and adolescents (mean age 9 years) hospitalized for COVID-19.	New-onset ODD present in 2.4% of patients.
Other infections		
Study and country of origin	Study design and sample description	Results
Parent et al., 2019 Canada	Cohort study of 13,204 children followed up till age 15.5 years and assessed for a history of recurrent gastroenteritis in the first 3 years of life.	Recurrent gastroenteritis in the first 3 years of life associated with a 7-9% increased risk of DBDs, particularly ODD. No association between levels of IL-6 and CRP measured at 9.5 years and DBDs at 15.5 years.
Maggi et al., 2024; Breitschwerdt et al., 2025	Case series of six persons with comorbid zoonotic *Bartonella* and *Babesia* infection, including one child aged 10.5 years.	New-onset ODD seen in the infected child; initially diagnosed as PANS before confirmation of dual infection.

ASO, anti-streptolysin O; CD, conduct disorder; CD4; cluster of differentiation 4; CRP, C-reactive protein; DBD, disruptive behavior disorder; HIV, human immunodeficiency virus; IL-6, interleukin-6; NK, natural killer; ODD, oppositional defiant disorder; OR, odds ratio; PANS, pediatric autoimmune neuropsychiatric syndrome; RNA, ribonucleic acid.

### 4.1 Immune-inflammatory dysregulation

The association between DBDs and PANDAS/PANS suggests that it is the immune response triggered by pathogens, and not the pathogens themselves, that is responsible for the emergence of symptoms of irritability, oppositional behavior, and aggression in these children. There is a relative paucity on studies of inflammatory markers in children and adolescents with DBDs, particularly in comparison to assays of neuroendocrine parameters (Goulter et al., 2025). Early research on immunological alterations in youth with DBDs yielded negative results: youth with these conditions did not exhibit significant differences in total leukocyte count, B- or T-cell distribution, or natural killer (NK) cell activity from those with depression or from healthy controls (Targum et al., 1990; Birmaher et al., 1994). Subsequent research found evidence of subtle immune dysfunction, including an increased plasma IgG3:IgG4 ratio (Pajer et al., 2002) and IgG autoantibodies that reacted against adrenocorticotrophic hormone (ACTH) (Schaefer et al., 2013). Studies of children with ADHD and comorbid DBDs have found that levels of high-sensitivity CRP (hsCRP) were correlated with symptoms of ODD, while levels of myelin-associated glycoprotein (MAG) were correlated with symptoms of CD (Tezcan et al., 2025). The cytokine IL-13 was also positively correlated with CD/ODD symptoms in this population (Gonzalez-Villen et al., 2024). Other markers of peripheral inflammation, such as white cell counts and ratios, do not appear to be associated with DBDs in children and adolescents (Seizer et al., 2025).

Despite several findings suggestive of subtle immune dysfunction in children with DBDs, it is not yet possible to link these to the effects of a given infection. However, it is worth noting that certain viral respiratory infections can, in principle, induce the formation of anti-ACTH antibodies, as have been reported in children with CD symptoms (Wheatland, 2004). In addition, animal models of PANDAS/PANS have found evidence of IgG antibody deposition in the corpus striatum associated with behavioral and motor changes; this may be related to the alterations in IgG ratios seen in some children with DBDs (Wald, 2019). How such changes may relate to possible infection-induced CD or ODD is unknown.

Furthersupport for the role of immune dysregulation in post-infectious DBDs comes from a recent case series of ten children with PANS following COVID-19 infection. After treatment with monthly oral prednisolone for three months, these children showed significant improvements in overall DBD symptoms, ODD symptoms, and CD symptoms. Though this result should be interpreted with caution, it suggests that immune modulation may aid the resolution of post-infectious DBD symptomatology (Berloffa et al., 2023).

### 4.2 Basal ganglia circuit dysfunction

Altered functioning of basal ganglia circuitry, particularly of connections between the frontal cortex and the caudate nucleus and striatum, has been implicated in the pathogenesis of childhood DBDs (Zhang et al., 2015; Pu et al., 2017). It has been suggested that complex psychiatric presentations in children, who exhibit symptoms of DBDs in association with obsessive-compulsive or ADHD symptoms, may be the result of basal ganglia dysfunction triggered by bacterial or viral infection (Yaryura-Tobias et al., 2003). This hypothesis aligns with some of the data from patients with CD or ODD occurring in relation to PANS, as discussed above. Some of the viruses associated with symptoms of DBD are known to cause basal ganglia lesions (Azab and Azzam, 2021; Hu and Cheng, 2024), but such lesions have not yet been demonstrated in children experiencing CD or ODD symptoms following infection.

### 4.3 Gut dysbiosis and altered gut-brain axis functioning

A third line of evidence suggests that certain infections may trigger conduct symptoms through alterations in the gut microbiome, which in turn leads to altered signaling in the gut-brain axis. Celiac disease, an intestinal autoimmune disorder, has been associated with high rates of ODD symptomatology in affected children (Amreen et al., 2025). Research in young children has found that specific alterations in the gut microbiome are associated with poor social skills (van de Wouw et al., 2024) and symptoms of DBDs (Nieto-Ruiz et al., 2023). Prenatal exposure to chemical toxins has also been associated with symptoms of CD, which appear to be correlated with altered gut microbial diversity (Zhou et al., 2024). In animal models of Parkinson’s disease, alterations in gut microbiota composition have been linked to changes in serotonergic and dopaminergic transmission in the striatum, suggesting that the microbiota-gut-brain axis can modulate the functioning of basal ganglia circuity (Ni et al., 2025). However, the relevance of such changes to the very different clinical scenario of childhood DBDs is debatable. More recently, some experts have suggested that both intestinal and extra-intestinal infections could trigger the onset of “pediatric disorders of gut-brain interaction” (p-DGBI). These conditions present with gastrointestinal symptoms, such as functional abdominal pain or irritable bowel syndrome (IBS) associated with symptoms of depression, anxiety disorders, ADHD, or ODD (Beinvogl et al., 2025). The exact association between p-DGBI and DBDs remains to be established.

## 5 A critical appraisal: correlation, causation, or confounding?

**Figure 1:**
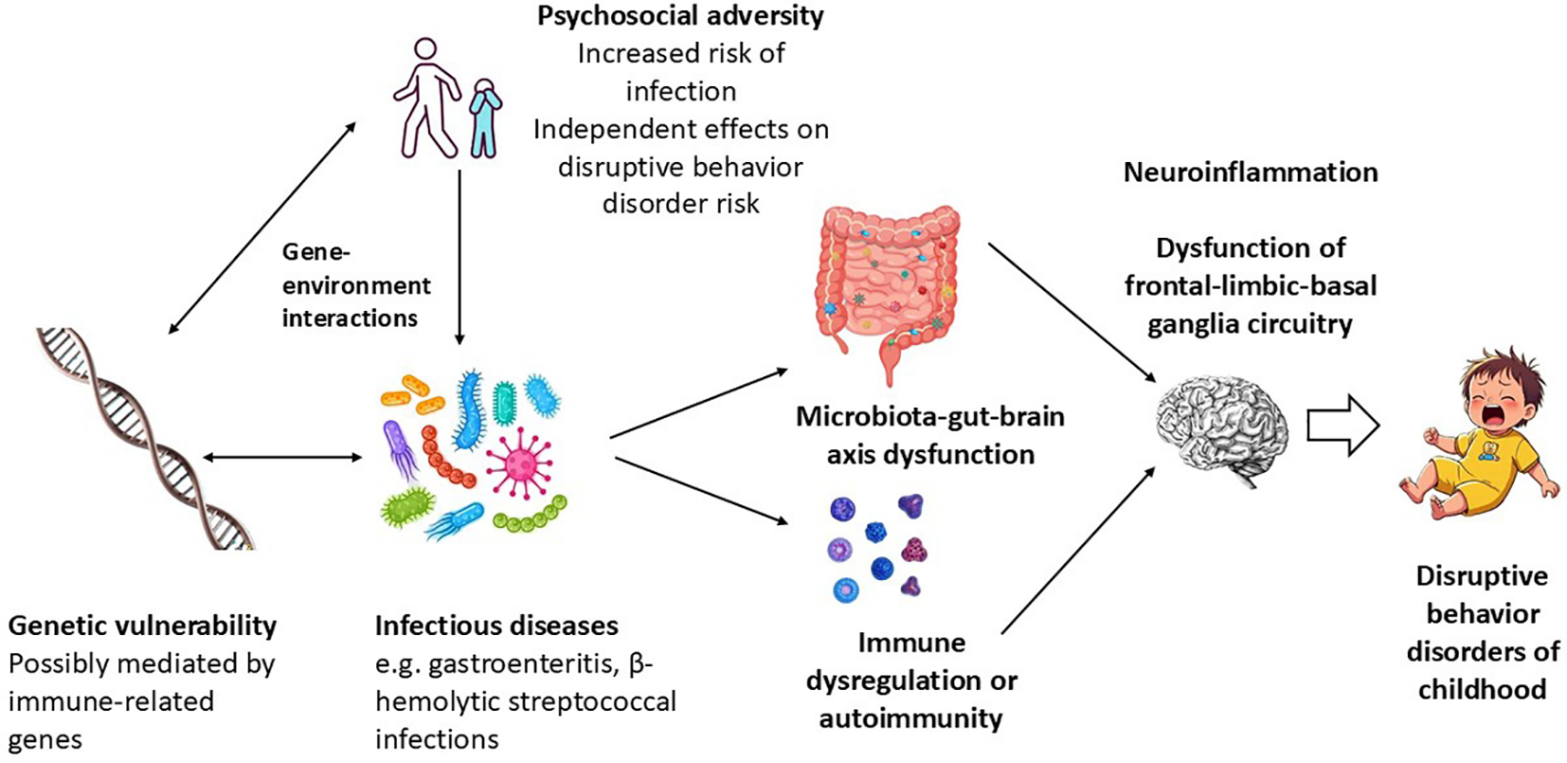
Possible mechanisms linking childhood infection to the emergence of disruptive behavior disorders in childhood.

The evidence summarized above suggests that an association between certain kinds of infection and DBDs is both biologically and epidemiologically plausible. The possible organisms and mechanisms implicated in this association are summarized in **Figure 1** below.

It must be acknowledged that the processes outlined in the above figure are speculative to some extent. The occurrence of high rates of DBDs in children with a particular infectious disease may indicate a causal link, but cannot prove it in the absence of a control group. It is possible that certain vulnerability factors may account for the co-occurrence of infection and CD/ODD without a causal link: for example, parental neglect can place a child at a higher risk of common respiratory or gastrointestinal infections, and is itself a risk factor for DBDs (Docherty et al., 2018). Another possibility is that infections account for only a limited proportion of DBDs, which arise due to gene x infection interactions. In this context, it has been suggested that a functional polymorphism of the interleukin-6 gene, though protective against childhood infections, can cause exaggerated peripheral and neuroinflammatory responses, leading to cognitive or behavioral impairment (Napolioni and MacMurray, 2016). Similarly, a recent family study suggests that there is a significant genetic contribution to PANDAS/PANS, which is associated with symptoms of ODD (Pol-Fuster et al., 2025).

A further intriguing possibility is that antibiotic therapy mediates the association between infections and DBDs. In two of the studies cited above, antibiotic therapy was used as a proxy marker for significant childhood infection, raising the question of whether the drug, rather than the infection, played a causal role in subsequent behavioral problems (Goodwin, 2011; Köhler-Forsberg et al., 2019). An independent study from Finland found that exposure to antibiotics either *in utero* or in the first two years of life was associated with a 1.2- to 1.5-fold increase in the risk of DBDs, even after adjusting for potential confounders. This result has been interpreted as suggesting that antibiotics interfere with the composition of the infant gut microbiome, leading to altered gut-brain axis signaling (Lavebratt et al., 2019).

Certain limitations of the available data must also be considered. First, in many studies, exposure to infection was also associated with an elevated risk of non-DBD psychiatric diagnoses in children, including anxiety, depressive, and obsessive-compulsive disorders (Rand et al., 2016; Köhler-Forsberg et al., 2019); the most specific association was with gastrointestinal infections (Parent et al., 2019). Second, many of the positive associations between infection and DBD have been reported in single samples but have not been replicated in independent settings. Third, few studies have examined the neural or immune correlates of CD or ODD symptoms arising in the context of infection. Fourth, it is difficult to test any of the above hypotheses in pre-clinical settings, because of the difficulties involved in developing a valid animal model of CD or ODD (Hernandez-Lallement et al., 2018): even if an animal model of, for example, gut dysbiosis in early life was developed, it is not clear which behavioral alterations could be taken as indicating a homologue of DBDs in human children or adolescents. Fifth, in cases where the exposure of interest was a severe infection in infancy, DBD symptoms may have been due to injury to the developing brain, rather than the direct effects of the infection or its resultant immune response. Finally, there is a scarcity of data on the efficacy of antibiotics or immunomodulators in DBDs related to acute, chronic, or recurrent infections. These limitations could be addressed through prospective clinical studies of children exposed to specific pathogens, or through the assessment of serological markers of infection in children with a recent onset of DBD symptoms. Further investigations of peripheral immune markers in children with DBDs, and their relationship with infection exposure, recovery, and DBD symptom scores, are also warranted (Goulter et al., 2025).

## 6 Conclusion

The hypothesis that infection during childhood increases the risk of disruptive behavior disorders is a promising one, with significant clinical and public health implications. This does not imply that infections “cause” DBDs: rather, they act in synergy with genetic and psychosocial vulnerabilities to trigger the emergence of these syndromes. The apparent association between infections and CD/ODD requires further exploration to confirm the existence of a truly causal relationship, to identify if such a relationship is a “class effect” or confined to specific pathogens, and to delineate underlying mechanisms, particularly those involving the immune system and gut-brain axis. From a clinical perspective, the confirmation of this relationship could facilitate better screening for symptoms of DBDs in children recovering from infections, leading to early diagnosis and more effective treatment, such as immunomodulatory therapies. It could also lead to modifications in antibiotic prescription practices, and to measures aimed at improving gut health, such as dietary modification or probiotics, in children who have suffered from gastroenteritis and exhibit disruptive behaviors. From a public health perspective, efforts to prevent the spread of childhood infections such as gastroenteritis could reduce the burden of DBDs at the population level, with beneficial consequences for families and communities. Such measures could form part of an integrated child health and development program, in conjunction with interventions aimed at reducing the psychosocial risk factors for disruptive behavior disorders.
